# Sagittal balance and intervertebral disc composition in patients with low back pain

**DOI:** 10.1590/1414-431X2022e12015

**Published:** 2022-11-11

**Authors:** L.G. Savarese, R. Menezes-Reis, M. Jorge, C.E.G. Salmon, C.F.P.S. Herrero, M.H. Nogueira-Barbosa

**Affiliations:** 1 Departamento de Imagens Médicas, Hematologia e Oncologia Clínica, Faculdade de Medicina de Ribeirão Preto, Universidade de São Paulo, Ribeirão Preto, SP, Brasil; 2 Laboratório de Pesquisa de Imagem Musculoesquelética, Faculdade de Medicina de Ribeirão Preto, Universidade de São Paulo, Ribeirão Preto, SP, Brasil; 3 Departamento de Física, Faculdade de Filosofia, Ciências e Letras de Ribeirão Preto, Universidade de São Paulo, Ribeirão Preto, SP, Brasil; 4 Departamento de Ortopedia e Anestesiologia, Faculdade de Medicina de Ribeirão Preto, Universidade de São Paulo, Ribeirão Preto, SP, Brasil

**Keywords:** Low back pain, Intervertebral disc degeneration, Magnetic resonance imaging, Spine, Radiography

## Abstract

The aim of this study was to verify the relationship between quantitative T2 relaxation measurements of lumbar intervertebral discs (IVDs) and spinopelvic parameters in patients with chronic low back pain. The study was approved by the Clinical Hospital of the Ribeirao Preto Medical School (USP) Ethics Committee, and written consent was obtained from all patients. A total of 455 IVDs from 91 consecutive patients with chronic low back pain were included in this prospective study. All subjects were assessed using the Oswestry Disability Index and visual analogue scale questionnaires and were confirmed to have no other spine diseases except disc degeneration. Spinopelvic parameters including the pelvic incidence (PI), pelvic tilt (PT), sacral slope (SS), sagittal vertical axis (SVA), global tilt (GT), T1 pelvic angle (TPA), lumbar lordosis (LL), thoracic kyphosis (TK), pelvic incidence minus lumbar lordosis mismatch (PI-LL), and lack of lumbar lordosis (LLL) were measured. The study group was categorized according to the Roussouly classification. Sagittal T2 maps were acquired to extract the IVD relaxation times, and the complete manual segmentation of IVDs at all levels was performed using Display^®^ software. Lumbar IVD T2 relaxation times showed significant correlation with PT (P<0.01), GT (P<0.01), TPA (P<0.01), PI-LL (P=0.01), and LLL (P=0.01). No difference was noted between Roussouly subtypes regarding T2 relaxation times at any disc level. Data from questionnaires showed no correlation with T2 relaxation times. Global tilt and T1 pelvic angle were correlated with IVD composition changes (T2 relaxometry). There was no correlation between clinical symptoms and IVD T2 relaxation times.

## Introduction

Degeneration of the intervertebral discs (IVD) has a high prevalence and is associated with low back pain (1-3). The evaluation of disc degeneration based on the Pfirrmann classification, which uses the T2-weighted magnetic resonance sequence, although useful, is subjective and limited in the assessment of early onset of IVD degeneration (4). The T2 relaxometry MRI technique is, otherwise, capable of quantifying and providing information on early biochemical changes in the IVD degeneration (5-9). Recent studies have established the relationship between T2 relaxometry and semiquantitative Pfirrmann classification, and the decrease in T2 relaxation times correlated significantly with increased disc degeneration (10-12). In addition, the relationship between sagittal balance and degeneration of the IVD using the semiquantitative Pfirrmann was demonstrated (13-16). However, we did not identify studies in the literature that evaluated the potential correlation between sagittal alignment and the biochemical composition of the IVD evaluated by quantitative magnetic resonance techniques. Given that previous studies revealed a negative correlation between T2 values and disc degeneration (6-8), we expected that patients with sagittal imbalance assessed through spinopelvic parameters would have more severe disc degeneration and consequently lower T2 values. Our hypothesis was that spinopelvic parameters indicating a sagittal imbalance are related with disc degeneration quantified by the T2 relaxometry technique. The purpose of this study was to verify the correlation between quantitative T2 relaxation measurements of IVD with spinopelvic parameters and clinical symptoms in patients with chronic low back pain.

## Material and Methods

### Patients

Ninety-one consecutive patients with chronic lumbar pain were included in this cross-sectional and prospective study. There were 56 female patients (mean age 53.5 years; range 23-76 years) and 35 male patients (mean age 53.6 years; range 19-73 years). Inclusion criteria were age greater than or equal to 18 years and chronic low back pain (visual analogue scale ≥3) with recurrent episodes in the last 6 months.

Exclusion criteria consisted of scoliosis or spondylolisthesis, vertebral fracture, neoplasia, postoperative status, disabling hip disease, infection, or bone metabolic diseases. Of the 107 evaluated patients, 23 were excluded for the following reasons: 10 individuals with spondylolisthesis, 5 individuals with scoliosis, and 1 individual with vertebral fracture. All individuals were assessed by the Oswestry disability index (ODI) and visual analogue scale (VAS). The study was approved by the Clinical Hospital of the Ribeirao Preto Medical School (USP) ethics committee, and all research was performed in accordance with the current regulations. Informed consent was obtained from all participants of this study.

### Spinopelvic parameters evaluation

Each patient underwent lateral panoramic radiography acquired with a CR Long Length Vertical Imaging System (Kodak Direct View, Carestream Health, USA). Subjects stood with arms supported on a stand, shoulders at 30° of flexion, and elbows slightly flexed according to previous literature (17) to minimize possible postural compensations.

**Figure 1:**
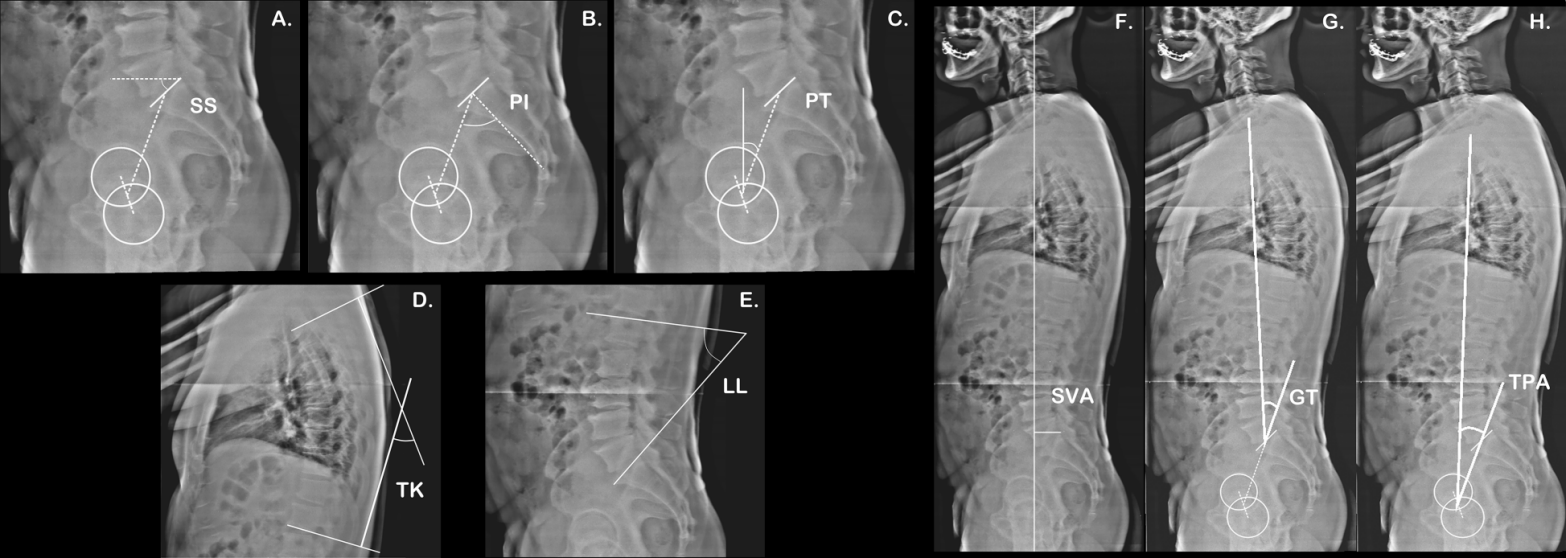
Panoramic radiograph of a 65-year-old male patient illustrating the spinopelvic parameter measurements: **A**, sacral slope (SS); **B**, pelvic incidence (PI); **C**, pelvic tilt (PT); **D**, thoracic kyphosis (TK); **E**, lumbar lordosis (LL); **F**, sagittal vertical axis (SVA); **G**, global tilt (GT); **H**, T1 pelvic angle (TPA).

Surgimap^®^ software (Nemaris Inc., USA, version 2.2.9.6) was used to measure spinopelvic parameters and vertebral curvature angles. The following parameters were evaluated (Figure 1): pelvic incidence (PI), pelvic-tilt (PT), sacral slope (SS), sagittal vertical axis (SVA), global tilt (GT), T1 pelvic angle (TPA), lumbar lordosis (LL), thoracic kyphosis (TK), pelvic incidence minus lumbar lordosis mismatch (PI-LL), and lack of lumbar lordosis (LLL). We used the formula proposed by Schwab and co-workers (18) to determine ideal LL, where LL = PI + 9. Therefore, using measured LL and ideal theoretical lordosis, we can establish the LLL of each patient. The contours of the femoral heads were marked, and lines were drawn adjacent to the sacral cartilage, at the upper plateau of the L1 vertebra, the lower plateau of T12, the upper plateau of T4, the upper plateau of T1, and the center of C7 and C2 vertebral bodies. From these markings, the software automatically calculated the spinopelvic parameters and the vertebral curvatures.

### Disc composition evaluation

We evaluated lumbar IVD composition using a 1.5T MRI scanner (Achieva; Philips Healthcare, Best, The Netherlands) with a 16-channel spinal coil. To avoid possible physiologic daily variations in disc water content, MRIs were acquired in the afternoon. All images of the lumbar spine were acquired in the sagittal plane using the following parameters: field of view=22×22 cm, thickness=4 mm, number of slices=16, and matrix=220×217. A 2D fast spin-echo T2-weighted sequence, echo time (TE)=120 ms, and repetition time (TR)=3900 ms were used for anatomical reference and IVD segmentation. We used a T2 relaxometry multi-echo sequence as follows: TE=20/40/60/80/100/120/140/160 ms and TR=3900 ms.

We generated T2 relaxometry maps using MINC tools and Display^®^ software (McConell Brain Imaging Centre, Canada) to extract the IVD relaxation times. T2 maps were computed on a pixel-by-pixel basis using an exponential decay model, where S0 is the equilibrium magnetization signal and S(TE) is the signal acquired with the echo time (TE):

$$S(TE)\;=\;S)\;*\;\exp\;\left(–\frac{TE}{T2}\right)$$

**Figure 2:**
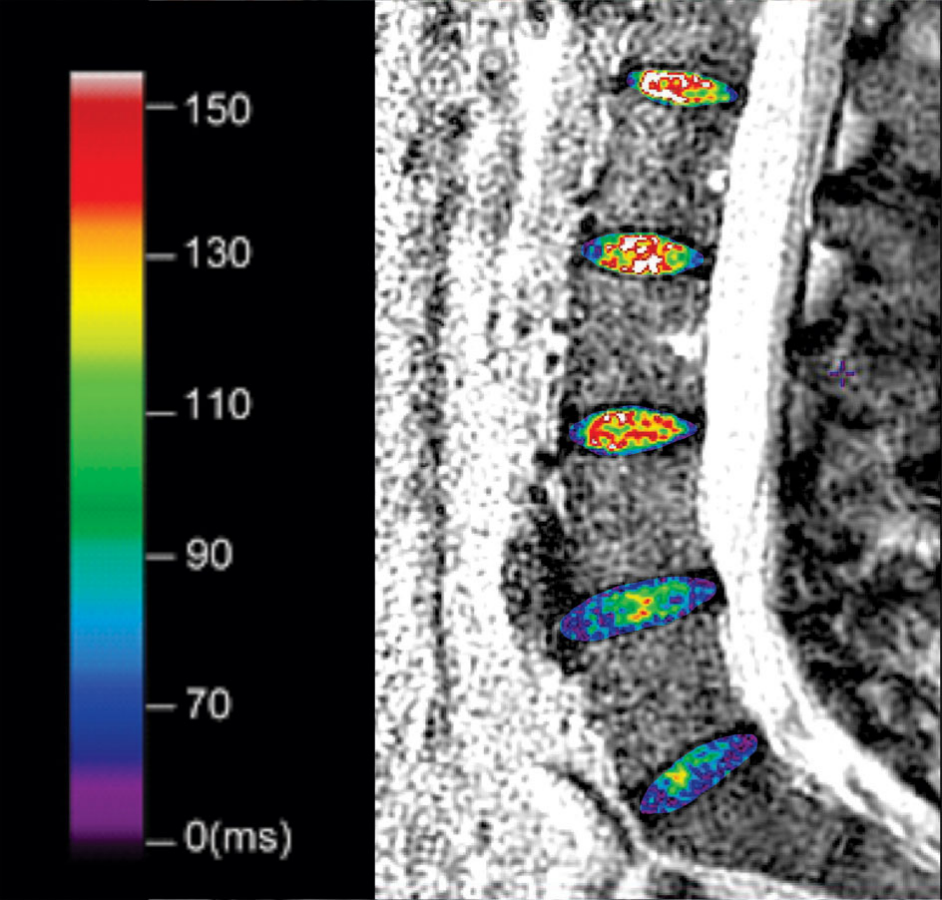
Image obtained from a 25-year-old woman with low back pain. Graphical representation of segmentation, encompassing the nucleus pulposus and annulus fibrosus of each lumbar intervertebral disc, with overlapping relaxometry maps. The schematic color scale represents the T2 relaxation times in milliseconds (ms).

Using T2-weighted images, the complete volume of each lumbar IVD was manually segmented (Figure 2). The segmentation encompassed both the nucleus pulposus and annulus fibrosus using all the sagittal images in which the five discs were identified, being careful not to include the subchondral bone.

### Image evaluation

The first examiner, a radiologist with four years of experience, was responsible for spinopelvic measurements and IVD segmentations. The second examiner, a researcher with four years of experience in spinal imaging who was blind to the measurements of the first examiner performed the same measurements to assess interobserver reliability. Participants were categorized into study groups by the two examiners according to the Roussouly classification for the 4 postural subtypes (19). When disagreement occurred, the opinion of a third examiner, a senior researcher and radiologist with more than 17 years of experience in musculoskeletal radiology, was considered. The distribution of the Roussouly subgroups was as follows: Type 1, n=13; Type 2, n=23; Type 3, n=44; and Type 4, n=11.

### Statistical analysis

Student's *t*-test was used to compare the composition of the IVD according to gender. We performed a mixed effects linear regression between the spinopelvic parameters PI, PT, SS, SVA, GT, TPA, LL, TK, PI-LL, LLL and the T2 relaxation times, controlling for the effect of age. Roussouly subtypes and T2 relaxation times were compared with analysis of variance (ANOVA). The correlation between questionnaire data and T2 relaxation time was analyzed using the Spearman correlation coefficient (ρ). The interobserver agreement was analyzed by intraclass correlation coefficient with a 95% confidence interval, considering zero to 0.19 as poor agreement, 0.20-0.39 as fair agreement, 0.40-0.59 as moderate agreement, 0.60-0.79 as substantial agreement, and 0.80-1.00 as almost perfect agreement. All statistical analysis was performed with a statistical software (SAS 9.2; SAS Institute, USA). For this study, a significance level of 5% was adopted. The statistical power (1-β error probability) of our sample (91 subjects) was 0.86 with an α error of 5%.

## Results

Table 1 presents the values of T2 relaxation times of the five IVD levels as means and standard deviations. The L2-L3 and L3-L4 discs presented the highest values for T2 relaxation times, and these values decreased as they approached the upper (L1-L2) and lower (L4-L5 and L5-S1) ends.

**Table 1:** T2 relaxation times of patients with chronic low back pain for each disc level.

Variables	Mean	Standard deviation	Minimum	Median	Maximum
L1-L2	91.4	12.0	69.0	91.0	131.4
L2-L3	92.5	10.7	72.4	92.4	127.7
L3-L4	92.1	11.1	71.8	89.8	119.3
L4-L5	91.8	11.5	69.9	89.8	132.2
L5-S1	90.2	11.4	69.9	88.5	129.8
Mean	91.6	8.9	75.7	91.0	114.5

Data are reported as mean±SD, minimum, median, and maximum.

The mean T2 relaxation time was T2=91.3±8.24 ms for men and 91.83±9.41 ms for women. No statistically significant differences in T2 relaxometry values were noted between genders at any disc level.

The spinopelvic parameters of our study group are provided in Table 2. Age was negatively correlated with T2 relaxation time in all segments (L1-L2: R: -0.49, P<0.01; L2-L3: R: -0.48, P<0.01; L3-L4: -0.26, P<0.01; L4-L5: -0.28, P<0.01; L5-S1: -0.20, P<0.01). A negative relationship was noted between T2 relaxometry values and spinopelvic parameters PT (beta=-0.26, P<0.01), GT (beta=0.25, P=0.01), TPA (beta=-0.31, P<0.01), PI-LL (beta=-0.18, P=0.01), and LLL (beta=-0.18, P=0.01) (Table 3).

**Table 2:** Spinopelvic parameters of patients with chronic low back pain.

Variable	Mean	Standard deviation	Minimum	Median	Maximum
PT(°)	14.7	8.2	-8	15	36
PI(°)	50.6	12.1	20	50	81
SS (°)	36.5	8.9	5	37	62
SVA (mm)	8.0	31.3	-78.6	5.1	103.3
GT (°)	14.7	9.6	-17	15	35
TPA (°)	10.4	7.6	-14	11	24
LL(°)	53.8	13.5	6	54	93
TK(°)	38.1	12.8	8	38	83
PI-LL(°)	-3.3	11.8	-46	-3	22
LLL(°)	5.7	11.8	-37	6	31

Data are reported as mean±SD, minimum, median, and maximum. PI: pelvic incidence; PT: pelvic tilt; SS: sacral slope; SVA: sagittal vertical axis; GT: global tilt; TPA: T1 pelvic angle; LL: lumbar lordosis; TK: thoracic kyphosis; PI-LL: pelvic incidence minus lumbar lordosis mismatch; LLL: lack of lumbar lordosis.

**Table 3:** Correlation between spinopelvic parameters and T2 relaxation times of lumbar discs, controlling for the effect of age.

Variables	Beta	P-value	95%CI
PI	-0.13	0.06	[-0.27; 0.01]
PT	-0.38	<0.01^*^	[-0.58; -0.18]
SS	0.07	0.49	[-0.12; 0.25]
SVA	0.03	0.21	[-0.02; 0.09]
GT	-0.26	<0.01^*^	[-0.44; -0.08]
TPA	-0.31	<0.01^*^	[-0.54; -0.09]
LL	0.03	0.68	[-0.10; 0.15]
TK	0.05	0.45	[-0.08; 0.18]
PI-LL	-0.18	0.01^*^	[-0.33; -0.04]
LLL	-0.18	0.01^*^	[-0.33; -0.04]

* P<0.05. PI: pelvic incidence; PT: pelvic tilt; SS: sacral slope; SVA: sagittal vertical axis; GT: global tilt; TPA: T1 pelvic angle; LL: lumbar lordosis; TK: thoracic kyphosis; PI-LL: pelvic incidence minus lumbar lordosis mismatch; LLL: lack of lumbar lordosis.

No difference was noted between Roussouly subtypes regarding T2 relaxation times at any disc level.

The mean ODI was 23.86±9.41 points (minimum: 5, maximum: 40, median: 25) and the mean VAS value was 7.48±1.88 (minimum: 2, maximum: 10, median: 8). No statistically significant correlation was noted between T2 relaxation values and questionnaire data at any disc level.

The ICC for T2 relaxometry values demonstrated almost perfect inter-observer agreement for manual disc segmentations (ICC: 0.96) and for spinopelvic parameters (ICC: 0.85 to 0.96) (Table 4).

**Table 4:** Intraclass correlation coefficients (ICC) and 95% confidence intervals (CI) of manual disc segmentation and spinopelvic parameter measurements.

	ICC	95%CI
Segmentation of the disc (T2)	0.96	[0.94; 0.97]
GT	0.86	[0.75; 0.92]
PT	0.90	[0.82; 0.94]
PI	0.87	[0.76; 0.93]
SS	0.85	[0.72; 0.91]
PI-LL	0.91	[0.83; 0.95]
LL	0.94	[0.89; 0.96]
TK	0.92	[0.86; 0.96]
SVA	0.96	[0.93; 0.98]
TPA	0.87	[0.76; 0.93]
LLL	0.91	[0.84; 0.95]

PI: pelvic incidence; PT: pelvic tilt; SS: sacral slope; SVA: sagittal vertical axis; GT: global tilt; TPA: T1 pelvic angle; LL: lumbar lordosis; TK: thoracic kyphosis; PI-LL: pelvic incidence minus lumbar lordosis mismatch; LLL: lack of lumbar lordosis.

## Discussion

This study assessed the correlation between quantitative T2 relaxation measurements of lumbar intervertebral discs and spinopelvic parameters reports using a consecutive series of 91 patients. We observed a significant negative relationship between T2 relaxometry values and the spinopelvic parameters PT, GT, TPA, PI-LL, and LLL. Roussouly subtype did not correlate with T2 relaxation time. We did not observe a significant correlation between mean T2 relaxometry values at the IVDs and questionnaire data.

Patients with lumbar degenerative disease are characterized by anterior sagittal imbalance, loss of LL, and increased PT (12,15,20,21). Ogon et al. (16) found an association between anterior annulus fibrosus degeneration in all lumbar disc levels and hypolordosis of the lumbar spine, anterior translation of the trunk and posterior inclination of the pelvis in chronic low back pain using quantitative MRI. SVA and PT are spinopelvic parameters that indicate the severity of adult spine deformity, but some points should be considered.

First, the SVA measurement can be reduced by postural compensatory mechanisms, such as pelvic retroversion. Therefore, a high PT may “hide” a larger spinal deformity when only the SVA is considered. SVA and PT are related and the magnitude of one affects the other. In patients who may have developed pelvic retroversion (high PT) to compensate for an underlying spinal malalignment and maintain their head over the pelvis (low SVA), the evaluation of the sagittal alignment by SVA alone would not detect their sagittal malalignment. Lafage et al. suggested that PT should be considered together with SVA to detect patients with spinal deformity in the sagittal plane without high SVA due to pelvic compensation (22). Of note, a successful realignment plan should not only restore the spinopelvic relationship but also “zero out” compensatory mechanisms, which drain energy and affect patients’ quality of life. Our results without relationship between SVA and T2 relaxometry reinforces that SVA should not be assessed alone in the evaluation of sagittal plane. In contrast, TPA and GT have advantages in assessing global alignment given that they account for pelvic retroversion and trunk anteversion and are not affected by postural or radiographic calibrations (23,24). Furthermore, they are strongly correlated with SVA, PT, and PI-LL (25) and showed relation with disc degeneration quantitatively assessed by T2 relaxometry.

The purpose of sagittal misalignment correction surgeries is to achieve a good global sagittal alignment and restore the LL, considering the morphology of the pelvis.

We observed no relationship between LL and disc degeneration quantified by T2 relaxometry. However, we identified the individual's LLL calculated by the difference between the observed lordosis and the ideal lordosis estimated by the Schwab formula. We also identified a relationship between the PI-LL, a valuable tool in the intraoperative planning (26,27), and T2 relaxometry values. These findings are expected when we consider the relationship between pelvic morphology, represented by PI, and LL. As postulated, LL has a strong correlation with PI; a high PI is accompanied by a high LL and a low PI is accompanied by a low LL (28). Thus, when two patients have the same LL and different PIs, the LL can be normal or reduced, depending on the PI value, and the calculation of the LLL provides this information. Therefore, individuals with a greater mismatch between pelvic incidence and lumbar lordosis (PI-LL) and higher LLL estimated by the difference between the expected lordosis and the observed lordosis, exhibited lower T2 relaxometry values in our study, indicating discs with more severe degeneration.

Consistent with other studies (29-31), we observed a negative linear correlation between age and T2 values. As older individuals tend to have more dehydrated discs and therefore lower T2 relaxometry values, we used a mixed effects statistical model that included age as a fixed effect. By fixing the age, we were able to evaluate the influence of spinopelvic parameters on disc degeneration without the bias of age. With that, we identified a significant negative correlation between the parameters PT, GT, TPA, PI-LL, LLL, and T2 values.

Patients with different Roussouly subtypes did not present significant differences in T2 relaxation times of IVD in our study. Of note, the classification of Roussouly et al. (32) was originally described in 160 asymptomatic individuals aged between 18 and 48 years. This classification system has its limitations given that individuals with low SS also tend to have low LL if they remain sagittally balanced in relation to the lumbar segment of the spine. Torrie et al. observed no significant correlation between postural Roussouly subtypes and semiquantitative Pfirrmann grading of disc degeneration (13). Consistent with this finding, our results reinforced that there is no correlation between Roussouly subtypes and disc degeneration in patients with chronic low back pain. A possible explanation is the fact that this classification is based on the SS and LL parameters, which exhibited no correlation with the T2 relaxometry values in our study.

We observed higher T2 relaxation values in the L2-L3 and L3-L4 discs and these values decreased in the upper (L1-L2) and lower (L4-L5 and L5-S1) lumbar spine extremities. It is reasonable to speculate that the L1-L2, L4-L5, and L5-S1 levels offer greater movement in the sagittal plane compared with other levels, given that L1-L2 is closer to the thoracolumbar junction. In addition, L4-L5 and L5-S1 support the largest amount of load due to gravitational force.

The T2 values found in the IVD were very close to those reported in the study by Niu et al. (29), who used ROIs that covered the nucleus pulposus and annulus fibrosus in the most central sagittal slice, and the study by Menezes-Reis et al. (33), who explored the segmentation of the vertebral disc throughout its extension. Most of the studies in the literature preferred to use the segmentation of NP and AF individually (10,30,34,35). Moreover, for practical reasons, most previous studies used small geometric ROIs for the evaluation of degenerated discs (11,34). Intervertebral discs classified as Pfirrmann I and II can be better distinguished when they are segmented separately. As degeneration progresses to Pfirrmann III and IV degrees, this distinction becomes difficult or impossible (36). Thus, a relative advantage considered in the choice of the segmentation method of this study was that the segmentation of the entire area or the complete volume of the disc allows a safer comparison between discs with different degrees of degeneration.

A study performed by Menezes-Reis et al. (37) with asymptomatic volunteers did not find an association between global spinopelvic parameters and IVD composition. Because their sample consisted of asymptomatic volunteers and the spine posture in these subjects could be considered within normal limits, a lack of association could be explained by the lack of individuals without sagittal plane imbalance.

There were some limitations in our study. First, our study used a cross-sectional design, and thus it is impossible to infer cause and effect relationships between the variables. Moreover, the study group consisted only of individuals with chronic low back pain, and asymptomatic individuals were not evaluated. Although the radiographic technique did not allow direct evaluation of lower limb compensation, systematic control was performed during the acquisition to prevent small compensatory mechanisms.

In conclusion, several spinopelvic parameters were correlated with disc degeneration quantitatively assessed using the T2 relaxometry technique. Given that this study involved the pathophysiology of mechanical association with disc composition, we believe that longitudinal studies with long follow-up are necessary to evaluate the potential future use of T2 relaxometry in clinical practice. To our knowledge, this is the first study correlating spinopelvic parameters GT and TPA with disc degeneration evaluated by T2 relaxometry.
